# Livestock/Animal Assets Buffer the Impact of Conflict-Related Traumatic Events on Mental Health Symptoms for Rural Women

**DOI:** 10.1371/journal.pone.0111708

**Published:** 2014-11-24

**Authors:** Nancy Glass, Nancy A. Perrin, Anjalee Kohli, Mitima Mpanano Remy

**Affiliations:** 1 Johns Hopkins University School of Nursing, Baltimore, Maryland, United States of America; 2 Center for Health Research, Portland, Oregon, United States of America; 3 Programme d′Appui aux Initiatives Economiques (PAIDEK), Bukavu, Democratic Republic of Congo; Univ of Toledo, United States of America

## Abstract

***Background*:**

In the context of multiple adversities, women are demonstrating resilience in rebuilding their futures, through participation in microfinance programs. In addition to the economic benefits of microfinance, there is evidence to suggest that it is an effective vehicle for improving health.

***Methods*:**

The parent study is a community-based trial to evaluate the effectiveness of a livestock microfinance intervention, Pigs for Peace (PFP), on health and economic outcomes with households in 10 villages in eastern Democratic Republic of Congo. The analysis for this manuscript includes only baseline data from female participants enrolled in the ongoing parent study. Multiple regression analysis was used to examine if livestock/animal asset value moderates the relationship between conflict-related traumatic events and current mental health symptoms.

***Findings*:**

The majority of women are 25 years or older, married, have on average 4 children in the home and have never attended school. Nearly 50% of women report having at least one livestock/animal asset at baseline. Over the past 10 years, women report on average more than 4 (M = 4.31, SD 3·64) traumatic events (range 0–18). Women reported symptoms consistent with PTSD with a mean score of ·2.30 (SD = 0·66range 0–4) and depression with a mean score of 1.86 (SD  = 0·49, range 0–3.47). The livestock/animal asset value by conflict-related traumatic events interaction was significant for both the PTSD (p = 0·021) and depression (p = 0·002) symptom models.

***Interpretation*:**

The study provides evidence of the moderating affect of livestock/animal assets on mental health symptoms for women who have experienced conflict. The findings supports evidence about the importance of livestock/animal assets to economics in rural households but expands on previous research by demonstrating the psychosocial effects of these assets on women's health.

***Trial Registration*:**

clinicaltrials.gov NCT02008708

## Introduction

Globally, the Democratic Republic of Congo (DRC) provides a distressing exemplar of the ways in which prolonged conflict, human rights violations, and the related health, economic, and social consequences can impact individuals, families, and communities. [1], [2] Humanitarian and emergency assistance programs focus significant resources on conflict and post-conflict settings, such as DRC, but the impact of these resources can be perceived as limited and resulting in unintended negative consequences for the population, particularly without intentional approaches for engaging affected populations and developing internal capacity to sustain successful initiatives.

In the Eastern DRC, men, women and children have been exposed to physical violence (women: 17·2%; men: 34·5%); movement violations such as abduction or forced displacement (women: 7·8%, men: 12·0%); and property violations (women: 23·6%; men: 30·7%) over a 16-year period (1994–2010) of conflict. [3] A population based survey in the accessible territories of North and South Kivu Province and Ituri District in Eastern DRC documented 39.7% (CI: 32.2–47.2%) of women reporting sexual violence with 74.3% (CI: 66.2–82.5%) of these survivors reporting sexual violence associated with the conflict. [3] Survivors are often further traumatized by extreme poverty, intimate partner violence (IPV), disease, loss of family and friends, stigma, and social isolation. [4], [5] A large body of research on trauma and mental health provides evidence for a consistent and often dose response relationship between traumatic events and symptoms related to post traumatic stress disorder (PTSD) and depression. [6]–[8] Yet, not all individuals who experience adversities exhibit long-term negative outcomes. The factors that mitigate or multiply the negative health, economic, and social situations created by conflict, however, are little understood. [9]–[11]

### Social upheaval as a tactic of war

Violence against civilians is used as a “deliberate and strategic tactic in war”, [4] to destroy or expel populations and pillage land and livestock. Most rural villages in the target area for this study have reported the loss of land and essential tools for farming resulting in limited agricultural productivity. [12], [13] Food insecurity in rural Eastern DRC has been well noted [14]–[16] [17] specifically that household members typically eat one meal a day and eat the same food every day (e.g., sweet potatoes, corn, cassava) with very limited access to protein from meat, eggs or milk. The looting or loss of animals (e.g. chickens, pigs, and cows) to raise, breed, and sell for funds to pay for household needs, school fees, and economic shocks (e.g. death of a family member) or opportunities (e.g. marriages, births) has further reduced household economic stability. [12], [18], [19] As agricultural production and animal husbandry has decreased in rural villages in the Eastern DRC in the past two decades of conflict, [12], [14] a cycle of food insecurity, [15] poor health, and extreme poverty [14], [16] has likely been further aggravated by exposure to traumatic events with limited access to quality health and social support systems. Even in the context of these multiple adversities, Congolese rural women and families are demonstrating resilience in rebuilding their futures, for example, through participation in microfinance programs. In addition to the economic benefits of microfinance, there is evidence to suggest that it may be an effective vehicle for improving health outcomes. [20]–[22]

### Congolese led livestock/animal microfinance - a way forward

Credit efforts, such as through microfinance initiatives, have demonstrated success in many communities and have resulted in an increase in household income to meet the food and nutrition, medical, and educational needs as well as reduce IPV in participating households. [20], [22], [23] Gaining access to and control over income-generating activities may also have positive benefits such as improved mental health because of the perceived ability by the head of household to meet the needs of the family, including educating children. [12] Many rural villages do not have access to sustainable financial services, such as banks or credit unions. In areas where financial institutions exist, they provide financial services with conditions that are inappropriate to the extreme poor, such as fees for basic services, high interest rates, and collateral for loans. As a result, poor families in rural villages are often unable to access credit to mitigate economic shocks, compounding their vulnerability to poverty. In particular, there is limited access by women to financial resources, as they often have no control over the household assets, such as property, necessary as collateral for credit in traditional financial institutions. [20], [22] Microfinance, defined as the provision of small loans (microcredit) to the poor to support their engagement in productive activities or grow small businesses, is becoming a mature industry in many parts of the world. However, it has remained primarily absent from rural areas and underutilized as an activity for households in rebuilding health for families in adverse situations. Households in rural villages used livestock/animals, including raising guinea pigs, rabbits, pigs, goats, and cattle as well as agriculture to sustain themselves and gain wealth in pre-conflict Eastern DRC, [12] therefore – focusing on the rural household's ability to raise and breed livestock/animals as well as use the livestock/animal waste for fertilizing crops is a potentially sustainable and culturally relevant microfinance approach in post-conflict, low resource settings, such as DRC.

The purpose of this manuscript is to provide evidence to support the use of livestock/animal microfinance as a sustainable approach to reduce the negative health outcomes, specifically post-traumatic stress syndrome (PTSD) and depression associated with multiple and prolonged exposure to traumatic events in women living in rural villages in low-resource, post-conflict settings. Specifically, the research question addressed is does livestock/animal assets buffer (or moderate) the effect of conflict-related traumatic events on symptoms consistent with PTSD and depression among women in rural South Kivu province of Eastern DRC? We also examined if the buffer effect remains after accounting for other forms of household wealth, such as household savings.

## Methods

### Study design and participants

In 2011, a randomized community trial was initiated to evaluate the effectiveness of a livestock microfinance intervention, Pigs for Peace (PFP), on health, economic and community-level outcomes with 10 villages in rural eastern South Kivu province. The protocol for this trial and supporting CONSORT checklist are available as supporting information (see Protocol S1 and Checklist S1). The trial is registered with ClinicalTrials.gov NCT02008708 in December 2013, one year after the start of enrollment of participants in the study. This delay in registration was an oversight by the lead author related to the challenging logistics of the research setting and the authors confirm that all ongoing and related trials for this drug/intervention are registered.

Prior to selection of the villages, introductory meetings were held between trained Congolese PFP research and microfinance agents (i.e., PFP agents) and village traditional and administrative leaders. Ten rural villages of Walungu Territory (located between 40–80 km from the capital of South Kivu province) were selected for participation in PFP based on: 1) feasibility of delivering a microfinance intervention over a wide geographic area with limited infrastructure; 2) commitment to the livestock microfinance approach and study by traditional chiefs and administrators; and 3) findings related to existence of other microfinance initiatives and social programs, village security and poverty from village-level assessments including review of administrative data and semi-structured interviews with key stakeholders. Adults, aged 16 years and older, were eligible for the study if they expressed a commitment to and understanding of microfinance principles (e.g. repayment of loan), were permanent residents of one of the ten participating villages and were responsible individuals in the household (e.g., married 16 year old, 16 year old responsible for younger siblings because of death of parent, widowed adult). Participation in the study was limited to one member (male or female) of a household.

### Livestock Microfinance Intervention

PFP is a collaborative microfinance program developed in partnership between Programme d′Appui aux Initiatives Economiques (PAIDEK), a Congolese microfinance organization, and the Johns Hopkins University School of Nursing (JHUSON). [13] PFP provides a loan to interested and committed households in the form of a 2 to 4 month-old female pig. The PFP microfinance approach uses pigs as a loan because livestock/animals are an important source of economic security and status, similar to a savings account, in rural villages and there are no cultural or religious taboos or gender-based responsibilities related to raising a pig. Each PFP member household cares for their pig including building a pigpen and providing adequate nutrition, health care and supervision with support from PFP agents. When the pig gives birth, members repay their pig loan in the form of two piglets, which are then used to provide new pig loans in the same village. After repaying their loan, the remaining piglets and the original female pig is the households to continue to raise, breed and/or sell. [13]

### Randomization

**Figure 1:**
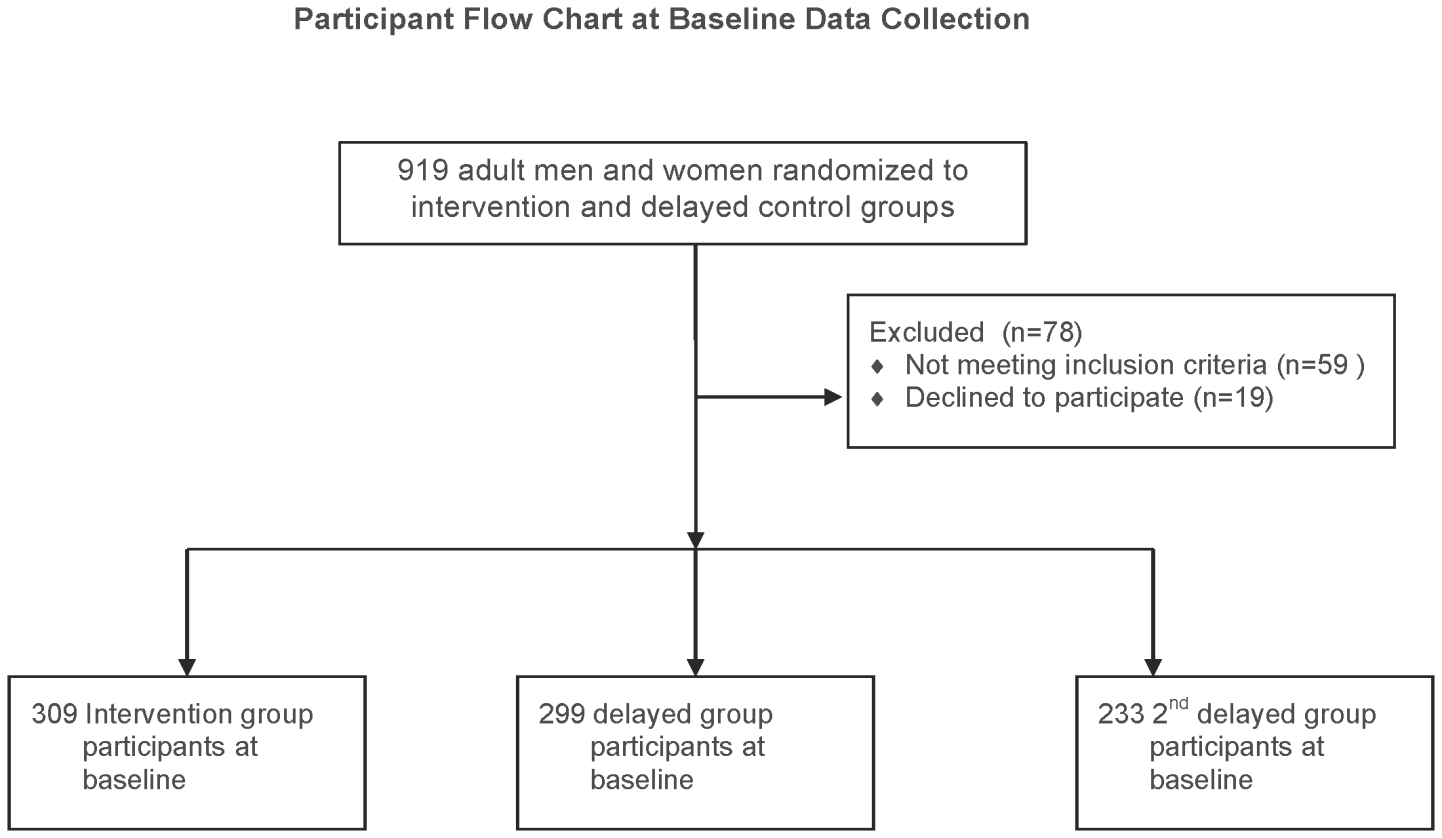
Participant Flowchart. Participant flowchart at baseline data collection.

A participatory village meeting facilitated by PAIDEK was held to discuss the PFP microfinance approach and study related activities over 24-month period. Community members interested in participation attended a second village meeting where an eligible household member was randomly assigned (see Figure 1), through a village lottery, to intervention (receive the initial pig loan) and delayed control group (receive the offspring repayment of the original pig loan). Due to high level of interest in PFP in the participating villages, a second delayed control group was formed such that a minimum of 66 households in each village were enrolled in the project (intervention and delayed control group) and the remaining households were placed in the second delayed control group (which also received a pig from the repayment offspring of the original loan).

### Data collection procedures

Baseline data collection reported in this manuscript took place after translation and back translation of the study questionnaire in French and local languages (Swahili and Mashi), pilot testing of questionnaire on tablet computers, and randomization, but prior to distribution of the female pig loan to the intervention group. The PFP questionnaire was developed using existing, validated research instruments and findings from this teams prior research. [13], [24] To address logistical challenges of conducting research in low resource settings, the team collected baseline data in two phases of five villages each between May and November 2012. Male and female PFP agents were trained as research assistants, with a focus on human subjects research ethics and safety for participants and PFP agents. As interviews were conducted when members would be earning their daily income, compensation for the time spent away, approximately 60-90 minutes, from work was provided as per local rates, approximately 1.50 USD.

### Ethics Statement

The Institutional Review Board [25] of the Johns Hopkins Medical Institute approved the study on 11/18/2010 (NA_00044037) and a committee of respected Congolese educators at the Universite Catholique at Bukavu (UCB) (due to lack of local ethics review board) reviewed and approved of the study, including risks and benefits to participants. Pilot and study interviews were initiated only after receiving oral, voluntary informed consent from the participant. Oral consent was approved during ethics review as the majority of our participants have never attended school, so written consent was a significant challenge. Further, inclusion of minor participants ages 16–17 years was approved during ethics review, if the minor reported he/she is married, widowed, parent or head of household. Study identification codes and names were recorded during one-on-one interview; all data recorded through the tablet-based program was uploaded to a password-protected server managed by the study team. Names were centrally removed and stored in a separate file.

### Study Questionnaire

For the purpose of this paper, we focus on measures in the baseline PFP questionnaire.

#### Demographics and Household Wealth

Based on the household survey developed by the IMAGE study team in South Africa [23] and WHO Domestic Violence and Health (2005) study [26], we collected demographic information on household members, including number of adult (16 years and older) and children by age and gender living in the household, marital status, education level, regular work (yes/no), perceived household wealth in comparison to other households in the village (i.e. 1 =  worse than others, 2 =  same as others, 3 =  better than others), dwelling details (yes/no for durable housing defined as roof made of tin, walls of wood/brick), and household savings (yes/no).

#### Livestock/animal Assets

The team surveyed nine livestock/animal vendors in five different village markets in the targeted study area and collected the current price to purchase the most commonly owned livestock/animal assets in the area. The average cost in US dollars are cows $450; pigs $70, goats $50, poultry $10, rabbits $8 and guinea pig $1. We computed a total livestock/animal asset score for each female participants household by multiplying the average market price for the livestock/animal by the number of household livestock/animals reported at baseline interview.

#### Traumatic Events and Mental Health

The exposure to trauma events section of the questionnaire was adapted from the Harvard Trauma Questionnaire (HTQ), a multi-part cross-culturally validated instrument that measures traumatic events and post-traumatic stress disorder (PTSD) [27]. Participants were asked about their exposure to 18 different traumatic events (e.g., deprivation, combat, forced isolation, sexual violence/humiliation) over the past 10 years. Exposure to trauma was analyzed as a continuous variable (0–18 different traumatic events). A 16-item version of section four of the HTQ [27] was used to identify symptoms consistent with PTSD in the past 7 days. The depression component of the Hopkins Symptom Checklist (HSCL) was used for reporting the experience of symptoms that bothered or distressed the respondent in the past one month. [27] An average symptom score for PTSD and depression was calculated. Both the HTQ and HSCL have been used widely and both have strong psychometric properties for the measure of traumatic events, and symptoms consistent with PTSD and depression in conflict-affected settings. [8], [28]–[30]

### Statistical Analysis

The analysis for this paper includes female participants who experienced trauma (n = 649; 77.4% of study participants). Multiple regression analysis was used to examine if livestock/animal asset value moderates the relationship between conflict-related traumatic events and current mental health status, specifically symptoms consistent with PTSD and depression. Livestock/animal asset value and conflict-related traumatic events in past 10 years along with their interaction were entered into the model. Two analyses were conducted, the first used PTSD as the outcome and the second used depression as the outcome. To determine if the moderating effect of livestock/animal asset remained after accounting for other indicators of household wealth, we included other measures of economic status (i.e. savings, perceived wealth, durable housing, and regular work) as covariates. In addition we tested perceived wealth relative to others in the community as a moderator of the relationship between traumatic events and current mental health symptoms.

## Results

### Sample Description

There were 705 female participants in the PFP microfinance parent trial, 92.1% experienced trauma. Baseline data collected from 649 female participants who had experienced trauma is reported. Table 1 provides the characteristics of the participants. The majority of women are 25–44 years (n = 335, 51.6%) followed by 45 years and older (n = 214, 32.9%) and 15–24 years (n = 100, 15.4%). Nearly three-quarters (70.7%) of participants are married, with 22.0% widowed. The median household size is 5 persons. Among those with children, the median number of children under the age of 18 years in the household is 4. Over 68% of women reported that they had never attended school. The majority of women (95%) reported that their home was made of non-durable materials (mud/clay); only 5.5% of houses were made of wood/brick. Village houses made of durable materials; such as wood/brick or tin sheets is a proxy of access to disposable income to purchase products to build durable homes. Further, less than 40% of households had roofing from durable material (e.g., tin sheets) with most women reporting living in homes with roofs made of dried leaves or grass. Few women reported that their household had savings (less than 5%) and less than one-quarter (23.0%) of women reported that they had regular paid work, such as being a porter or vendor in the market, domestic servant or farm hand. Almost 50% of women perceived that their household was worse off financially than other households.

**Table 1:** Participant Characteristics.

Demographics	
*Age (%)*	
15–19 years	1.4
20–24 years	14.0
25–34 years	30.0
35–44 years	21.6
45–60 years	27.7
Over 60 years	5.2
Currently married (%)	70.7
*Education (%)*	
No schooling	68.7
Never completed primary	15.9
Completed primary	13.9
Completed secondary or above	1.6
**Household wealth**	
Has a tin roof	37.9
Has wood or brick walls	5.9
Has savings (%)	4.8
Is currently working outside home and paid (%)	23.0
*Perceived household wealth relative to others in the community:*	
Worse than others	51.2
About the same as others	36.7
Better than others	12.1
**Traumatic Events and Mental Health**	
PTSD mean (SD)	2.30 (0.66)
Depression mean (SD)	1.86 (0.49)
Number of Traumatic Events (range 1–18)	4.31 (3.64)

N = 649.

### Livestock/animal asset value

Nearly 50% of women reported that her household owned guinea pigs (49.3%), 32.4% goats, 29.3% poultry, 12.6% cows, 15.1% pigs, and 2.2% rabbits. The household livestock/animal asset value score ranged from $0 to $1,912 and the distribution of the score was skewed so quartiles are used in the analyses. The first quartile included those with no animal assets. Participants in the second quartile (range $1–$20) reported household livestock/animal assets of a few guinea pigs, a rabbit or a couple of chickens. Only participants in the highest quartile (range $107–$7,912), although not all of them, reported household livestock/animals assets such as cattle.

### Conflict-Related Trauma Events and Mental Health

Of the 18 traumatic events, participants reported a mean of over four conflict-related traumatic events (M = 4.31, SD 3·64) on the Harvard Trauma Questionnaire (HTQ) in the past ten years such as being close to death, imprisonment, and/or witness/experience sexual violence/humiliation. Table 2 provides the percent of women experiencing each type of event. The most common events were lack of food, water, and medical care and being in a combat situation. Symptoms consistent with PTSD (e.g. recurrent thoughts of hurtful events, difficulty sleeping, trouble concentrating) in the past week were reported on a Likert scale with responses from never (1) to extremely (4) with a mean score of 2·30 (SD = 0·66). PTSD scores ranged from 0 to 4. Symptoms of depression (e.g. feeling low in energy/slowed down, blaming self for things, feeling worthless) in the past month were reported on a Likert scale ranging from not at all (1) to a lot (4); the mean score was 1.86 (SD  = 0·49). Depression scores ranged from 0 to 3.47. The correlation symptoms of depression and PTSD was.623 (p<.001).

**Table 2:** Percent of women experiencing conflict-related traumatic events in past 10 years.

Traumatic Event	% Female Reported
Ill health without access to medical care	68.3
Lack of food or water	63.5
Combat situation	52.9
Forced separation from family members	30.2
Unnatural death of family or friend	27.4
Lack of shelter	25.7
Being close to death	25.1
Murder of family or friend	22.7
Tortured or witnessed torture	20.2
Brainwashing	17.6
Serious injury	17.3
Witnessed rape or sexual abuse	12.3
Forced isolation from others	9.9
Rape or sexual abuse	8.6
Imprisonment	8.0
Lost or kidnapped	7.6
Murder of strangers	7.1
Other sexual humiliation	7.1

### Livestock/Animal Asset as a Moderator of Conflict-Related Trauma and Mental Health

Results of the regression analyses are summarize in Table 3. The livestock/animal asset value by conflict-related traumatic events interaction was significant for both the PTSD (p = 0·008) and depression (p = 0·002) symptom models. The interaction remained significant when controlling for other household wealth indicators (e.g. savings, durable housing, perceived wealth, regular work) for PTSD (p = 0·021) and depression (p = 0·002) symptoms. The perceived wealth by conflict-related traumatic events interaction was not significant for PTSD (p = 0·717) or depression (p = 0·140) symptoms.

**Table 3:** Regression Results Using Baseline Data from Parent Study.

	PTSD			Depression		
	Standardized Regression Coefficient	Std. Error	p-value	Standardized Regression Coefficient	Std. Error	p-value
Livestock/Animal Assets	.083	.035	.147	.153	.028	.013
Traumatic Events	.652	.017	<.001	.598	.014	<.001
Livestock/Animal Assets X Traumatic Events	−.247	.006	.021	−.358	.005	.002

Covariates in the model: Perceived wealth relative to others, having a tin roof, having durable walls, having savings, and currently working.

## Discussion

The study provides evidence of the moderating affect of livestock/animal assets on current mental health symptoms for women who have experienced prolonged conflict and multiple traumatic events. Specifically, as the livestock/animal assets increase, the impact of conflict-related traumatic events on symptoms consistent with PTSD and depression are reduced. The findings supports existing evidence about the importance of livestock/animal assets to economics in rural households but expands on previous research by demonstrating the psychosocial effects of livestock/animal assets on health. Livestock/animal assets extend beyond its association with wealth, as other measures of wealth used to assess household economic security (e.g., durable housing, savings, regular work) did not buffer the effect of conflict-related traumatic events on symptoms consistent with PTSD and depression for women. Women report using the funds gained through the livestock/animal microfinance to pay for school fees, purchase land and materials to build/repair homes, [13] thus potentially strengthening self-perception of status as well as the household's and larger communities perception of the woman's productivity and status. [13], [14]

In sub-Saharan Africa and DRC, household needs are often complex due to large family size and the support given to extended family, for example - the median household size in the current study is five people, with on average of 4 children under the age of 18 years, and women report frequent food insecurity, eating only one meal a day. In post-conflict settings such as DRC, the direct (e.g. physical and sexual violence) and indirect (lack of educational and health system) effects of conflict further impact a woman's productivity, health, economic security and status. In resource limited and conflict-affected rural milieu like DRC, community-based livestock/animal microfinance interventions present a culturally acceptable intervention that can simultaneously provide psychosocial support to address mental health needs for women while supplementing family economic security.

Most rural households globally rely on subsistence farming to ensure food security and animal husbandry to obtain wealth and status in the village. [31] In rural DRC, livestock production may continue to be one of the few economic opportunities available to villagers to accumulate needed resources to rebuild their household wealth and social status. [32] Livestock such as pigs are traditional rural assets, [12], [14], [19] and, therefore, not intended for regular food consumption but serve as a “savings account” for economic opportunities and crises. Livestock/animals are visible symbols of productivity and social status, and their possession influences the owners' positive perception of self and household wealth. [14], [19] As an animal that can be raised, bred and sold by women in DRC, pigs importantly represent a gender-neutral intervention in eastern DRC that can bring adult men and women together to improve social, health and economic outcomes for their family. [33]

To address unmet mental health care needs of a post-conflict setting, Bass and colleagues (2013) conducted a study with female sexual violence survivors in Eastern DRC to examine the effectiveness of an adaptation of group cognitive processing therapy (CPT) provided by community-based psychosocial assistants supervised by psychosocial staff at an international nongovernmental organization (NGO) and by US-based clinical experts. [34] The findings indicate that psychosocial assistants (lay health workers) with appropriate training and supervision can implement psychotherapeutic treatments such as CPT. The study provided impressive findings of the effect of CPT on mental health for sexual violence survivors, the sustainability of the CPT intervention approach is a challenge for low-resource, rural settings where women have experienced different and multiple conflict-related traumatic events.

In DRC, as in many low-resource countries, there continues to be a lack of government funded health centers with human resources that have training in psychotherapeutic treatment such as CPT to provide ongoing supervision and support for lay health care workers (e.g., psychosocial assistants). For example, it is estimated that DRC has 0.07 psychiatrists working in mental health sector per 100,000 population. [35] Further, DRC's approved mental health policy (passed in year 1999) does not allow primary care nurses to diagnose or treat mental health problems, so it is unclear if the role of the lay health care worker in the provision of services is an accepted practice by the DRC government and if accepted who will provide ongoing supervision in rural areas. [35] In addition, there does not appear to be an established funding source and training curriculum for the psychosocial assistants supported by the national or local government, as the WHO mental health atlas (2011) reported that the majority of primary health care doctors and nurses have not received official in-service training on mental health within the last five years and approved manuals on the management and treatment of mental disorders are not available in the majority of primary health care clinics. Although the evidence supports the potential of CPT to provide psychosocial support to survivors of sexual violence, expanding into rural areas with limited infrastructure will likely take significant, ongoing international resources that are becoming continually difficult to access given the multiple and protracted global conflicts. Further, existing CPT interventions are often focused on a singular traumatic event (e.g. sexual violence) and diagnostic criteria, and therefore evidence for scaling up the intervention to address the multiple traumatic events experienced by women over a prolonged conflict is unclear.

The PFP model, which was collaboratively developed by the target community, provides support for the importance of indigenous expertise in developing and implementing sustainable programs to improve mental health in post-conflict areas. PAIDEK's microfinance model of engaging traditional leaders and community members in productive and culturally appropriate economic activities that engage both men and women in rural areas, [13] where most international organizations do not have sustainable access or trained staff to provide ongoing health and economic programs, is critical to ending a dependence on international humanitarian aid and progress to long-term development initiatives that will advance wealth and status of women and rural household members, with the positive effect of buffering negative mental health symptoms. It is certain that livestock/animal microfinance alone will not solve the multiple economic, health and social challenges facing women and families in low resources conflict and post-conflict settings. However, integrating collaboratively developed and culturally relevant economic initiatives while strengthening health care and social systems in rural areas are critical steps towards sustainable development.

This study has several limitations. The cross-sectional analysis limits the teams' ability to determine causality. The analysis presented here provides initial evidence for a culturally acceptable village based livestock/animal microfinance intervention to buffer the mental health effects of traumatic events on women. Exposure to traumatic events represents a history of exposure within the past 10 years, representing the multiple periods of conflict in the Walungu Territory. Some of these experiences were likely to be in the recent past and others several years prior to interviews, so recall bias is a potential issue. In spite of these limitations, this study has particular strengths. Firstly, the innovative approach of promoting resilience in a situation of social upheaval, secondly, the integration of conceptual elements of addressing both health and economic security and thirdly the importance of partnering with a well-established and respected Congolese-led microfinance organization (PAIDEK) with an ability to engage in sustained partnerships with village leaders to implement PFP. This study has adopted both innovative conceptual and methodological processes to advance evidence to achieve scalability and sustainability to address social, cultural and economic factors associated with poor health for women in conflict-affected rural villages.

## Conclusions

Our findings contributes to the science base for large-scale implementation of sustainable, community-led animal/livestock economic programs that not only increase household wealth and status, but improve health in areas where women and other household members have extremely limited access to high quality health and social services.

## Supporting Information

Protocol S1 **Trial Protocol.**
(DOCX)

Checklist S1 **CONSORT Checklist.**
(DOC)

Data S1 **Dataset for this analysis.**
(SAV)
