# Supplementary material for: Livestock/Animal Assets Buffer the Impact of Conflict-Related Traumatic Events on Mental Health Symptoms for Rural Women
Source: PLoS One. 2014 Nov 24;9(11):e111708. doi: 10.1371/journal.pone.0111708 (PMC4242503; doi:10.1371/journal.pone.0111708)
Supplement: Protocol S1 — Trial Protocol. (DOCX) [file pone.0111708.s001.docx]

**PROTOCOL S1: Trial Protocol**

Title: Microfinance Intervention to Improve Health of Trauma Survivors in DRC

PI: Nancy Glass

Study Number: NIMHD R01 MD006075 (December 2010-November 2015)

Original Hopkins IRB approval on November 18, 2010

1. **Specific Aims**

Mobutu Sese Seko’s government of “Kleptocracy” collapsed in 1997 after 30 years of oppression. The new nation that emerged, the Democratic Republic of Congo (DRC), remains an all-to-potent reminder of how human rights violations, and their related health and economic impacts, can devastate individuals, families and communities. The genocide in neighboring Rwanda, coupled with the collapse of the Mobutu government, has spawned two wars and over a decade of warfare throughout the region, resulting in millions of deaths in what is the deadliest conflict since World War II^1^. The last decade has seen the use of rape as a weapon of war in the DRC, where rebels and soldiers subject men, women and girls to brutalizing attacks, rape, torture, and mutilation. Survivors of the assault are often further traumatized by infections, disease, poverty, stigma and social isolation.

To illustrate the impact of the war on the health and economic resources of individuals and villages in the DRC, the Chief of Ngweshe provided the following information on the Kaniola District (pop. 96,000) of the Walungu Territory, the focus area of the proposed study. From 2000-2007, 1,678 persons were killed; 1,554 people were kidnapped and held for ransom; and 4,354 women and girls were raped, of whom 217 were girls under the age of 15 years. In the same period, 25 schools and 12 health centers were destroyed, and 18,802 cows and 8,625 goats were stolen, eliminating educational and health care services and the economic livelihoods and food sources of local populations. The cases of rape are likely under-reported due to the stigma associated with rape and the fear of isolation from family and community. Families report not having money to pay for needed treatment and care for injuries and/or infections associated with the rape, or to transport the victim to a health center for care.

The US plays a significant role in global health. It is both the largest funder of innovation in global health and the largest donor to care and support programs in sub-Saharan Africa—notably through PEPFAR and responses to humanitarian crisis, such as USAID funded programs in DRC. The effectiveness and sustainability of these efforts are limited by gaps in knowledge of the role of social determinants, such as poverty, social isolation, chronic stress and trauma, and limited access to health care services has on the health of women and families.^9^ To begin to address these gaps, our overall goal is to build the science base for large-scale implementation of economic programs to improve the economic security and health in man-made and natural disaster settings. The objective is to test the effectiveness of an established innovative, village-led microfinance program, Pigs for Peace, on health, household economic stability, and reintegration to family and village. Our preliminary evidence indicates that a village-led animal husbandry microfinance program improves Congolese women’s health and household economic stability, through increased funds to purchase food, housing and medication and to pay school fees for children. Survivors report a reduction in the negative health impacts of chronic stress, stigma and trauma associated with trauma and violence because they are once again productive family and village members. Therefore, a five-year experimental trial will be conducted using mixed-methods to address the following aims:

**1. Determine the effectiveness of a village-led microfinance program on participant health and reintegration in intervention households compared to participants in delayed control households.** Health and reintegration will be measured at baseline and six, twelve, and 18-months post-baseline using self-report in both intervention and control groups. We hypothesize that at six, twelve and 18 months post-baseline participants in intervention households will have improved health and increased reintegration to families in comparison to control households.

**2. Determine the effectiveness of a village-led microfinance program on household economic stability in intervention households compared to delayed control households.** Household economic stability will be measured at baseline and six, twelve and 18 months post- baseline using self-report in both intervention and control households. We hypothesize that at six, twelve and 18-months post-baseline the intervention households will have improved household economic stability in comparison to control households.

**3. Examine the role of a village-led microfinance program on village-level health, economics stigma and reintegration of participants and their families in intervention and delayed control households.** Village members (n=5 in each village, n=50 total) will complete a baseline and 18 month post-baseline qualitative interview to examine the role of microfinance on village-level health, economics, stigma and reintegration in both intervention and control villages.

Violence, including torture and sexual violence is a global health issue and is a violation of human rights and has been used as a weapon of war in the DRC. The multiple interrelated and complex social determinants of health identified above, such as poverty, stigma, chronic stress and trauma, and limited access to health care services impacts the health of individuals, their household and village. As a major donor to global health and humanitarian crisis, the US needs improved evidence of sustainable community-led intervention models for improved health outcomes in low-income countries, including disaster settings. Therefore, the objective for this study is to test the effectiveness of an innovative, village-led microfinance program on health, household economic stability, and reintegration to family and village. The study will be conducted by Johns Hopkins University School of Nursing in collaboration with partners Great Lakes Restoration (GLR) and Programme d’Appui aux Initiatives Economiques du Kivu (PAIDEK) in the Chiefdom of Ngweshe in the Walungu Territory in South Kivu, DRC.

**Background: Pigs for Peace Livestock/Animal Microfinance Program**

The purpose of the village-led animal husbandry microfinance project, Pigs for Peace (PFP) is to improve the economic well being of men and women and their families living in villages in the rural areas of South Kivu in the eastern DRC and therefore, improve the health of the individual, households and villages. The focus is on providing a “loan” of a pig to women and their families as a means to generate household income. Why pigs? The experience of our collaborator, PAIDEK microfinance, indicated that rural villagers were not able to make repayment of money loans, due to their lack of income generation activities because of the looting of essential tools and animals by rebels and soldiers. Farming and breeding animals is primary source of food and income for rural households in the targeted area. Further, the trauma of the war and violence had limited productivity of household members, as men and women fear for their safety when working in the fields. Therefore, pigs were selected as they do not need a large amount of space to live and forage, reducing the need for men, women and children to travel far from the village to seek food and exercise the pigs, thus limiting their risk of encountering rebels and soldiers in the fields away from the village. The pigs eat everything and have been commonly raised in Congolese villages. Importantly, whereas men in the DRC usually control household decisions regarding the sale of goats or the family cow, women can make decisions about the purchase, sell and breeding of the family pig. PFP is a partnership between Great Lakes Restoration (GLR, www.glrbtp.org), Programme d'Appui aux Initiavives Economiques du Kivu (PAIDEK) and the Johns Hopkins University School of Nursing. All colleagues working on PFP have volunteered their time and effort towards its success.

PFP is simple and effective. A village agrees to join PFP and develops a village PFP association. An association in the villages supports the families in managing the pigs, from building enclosures, to vet services, to education on food and health of pigs, and how to bring others into the association. The village association agrees to meet monthly to discuss challenges and solutions as well as successes for the PFP project. The village association meetings have also become a source of ongoing strengthening of social capital and social network, where important health and human rights related information is shared. The village PFP association decides what member households in the village receives the female pig and the association delivers the pig to the selected village households with the female head of household the primary target audience. The project also provides adult male pigs to the village association for the initial breeding. Each participating household agrees to repay their “loan” by giving one piglet to the PFP association from the first two litters of piglets. These piglets are then given to other village households in the association, allowing PFP to grow rapidly and serve families in need of resources to sustain and rebuild their lives.

The pigs are providing sources of sustainable, supplemental income, with pigs giving birth frequently – 2 times a year with up to 6-12 piglets at each birth. In July-August, 2009, an initial PFP evaluation was completed with the first 25 village women who received the pig loan. The participants who had a pig give birth reported using funds from selling pigs ($20-$40 each) to pay for their children’s school fees, clothing, and medicine, to build a house, and/or to invest in other small businesses. This amount of household income is significant in this area, as some households report living on $1-$3 a day.^55^

PFP was implemented in December, 2008 with just 10 families in one village near Bukavu in South-Kivu province of the eastern DRC receiving “loan” piglets from the project. As of the end of 2009 these totals had expanded to 98 families in 14 villages. Counting children in the household the total number of individual beneficiaries of the program was 336. In addition 6 pigs have been provided to the *Flamme de l’amour* (Flame of Love) Orphanage located near Goma in North- Kivu. The PFP activities have been accomplished with total funds of $4,480 received from GLR at an average cost of nearly $46/pig. In addition, the Project has received the first 6 “interest” piglets from the participating households. Further evidence of the positive impact of the project on the ground in the DRC is the size of the waiting list; over 700 more families are prospective participants. Funding for Pigs for Peace has come from individual contributions and fund raising efforts by GLR and Johns Hopkins University (School of Nursing, Medicine, and Public Health) student organizations. Contributions are used to purchase pigs ($40) and provide assistance with transportation and education on pigs to the village association (up to $10). An annual report and newsletter of the PFP project is attached in Appendix A. The proposed study will provide an opportunity to expand the PFP project and experimentally evaluate the effectiveness of the microfinance intervention on the health, household economic stability and reintegration of trauma survivors in rural villages in Ngweshe Chiefdom in the Walungu Territory of South Kivu, DRC.

**APPROACH**

The study was developed by our collaborative microfinance-academic team that has been working together for the past 3 years to improve the health and well-being of participants and their families. Our demonstrated success in partnership, complementary qualifications of team members and preliminary findings has convinced the team to propose a five-year experimental trial using a mixed-methods (quantitative and qualitative) approach to successfully achieve the study aims. The following section describes the teams’ specific aims and approach in detail.

Design Overview for Specific Aims 1 & 2

Ten villages in the Walungu Territory in South Kivu, DRC will be selected through a village assessment. A total of 10 villages will be selected for participation. Sixty-six households in each village will then be randomized to intervention (receive 1^st^ loan pig) and control (receive 2^nd^ loan pig) groups. Outcomes will be measured at the individual and household levels in both intervention and control villages. Measurement of outcomes in each village will use self-report by village household members. An eligible household has at least one individual (16 years or older). The study will be conducted over five years. Intervention and control group outcome data will be collected at baseline and, 6, 12 and 18-months post-baseline assessment.

Study Setting:

The study will take place in the tribal chiefdom of Ngweshe, which is located in the Walungu territory, South Kivu, DRC. The Chiefdom is 40 km south of Bukavu, the capital city of South Kivu, and has an estimate population of 700,000 residents and was chosen by the research team and collaborating Congolese partners for several important reasons: 1) this rural area has suffered significantly from the war over the past 15 years because of its proximity to mineral resources (e.g. gold and coltan) and isolation related to limited infrastructure; 2) very limited humanitarian and/or development resources, including microfinance have reached the Chiefdom; and 3) the team has a strong history of collaboration (over 3 years) with Congolese physicians, nurses, agriculture technicians, community health workers, religious leaders and village leaders working in the Walungu Territory to better understand the needs of the villagers.

The number of villages included in the study was determined by the operational feasibility of delivering the intervention over a wide geographical area, the time required for recruitment and follow-up, the need to enroll all eligible households in a village before expanding, and ethical concerns about withholding participation from control villages. A planned 10 villages in the Chiefdom will be selected for the study. Randomization of 66 households that have expressed interest in the study will be done during a research team meeting. One household name will be drawn blindly and assigned to the intervention group and then the next assigned to control and so on until complete for each village. Before the study, no village will have access to microfinance or the PFP program. Participating village populations have 75-100 households, with typically over 50% of village population being female, because of the loss of boys and men to violence or leaving the village to find work to help support the family.

Phase One: Description of Village Assessment and Informational Meetings

In Phase One (Year 1), we will complete the following study related activities: 1) village assessment: the socioeconomic characteristics (health clinics, schools, markets, economic activities, etc) and the number of households in the village and the number of households with women ages 16 years or older in 10 potential study villages in the Chiefdom will be assessed through field reconnaissance surveys and interviews with village members; 2) the study team will then meet with village leaders (traditional and governmental) in the selected 10 villages in the Chiefdom to invite them to participate in the study. If a village decides not to participate after the initial meeting, the team will meet to select another village to invite based on the field assessments. The invitation meeting will be held in the villages with leaders to discuss the study goals, explain the study design, including an explanation of randomization and intervention and delayed control groups. The team and village leaders will organize the informational meeting and invite all village households to attend and participate in the discussion; and the team will recruit and train (study protocols, human subjects protection) one (female or male) research assistant to work with 2 village each.

*Informational Sessions:* In year one, the informational sessions for households in participating villages will include: 1) introduction to study team members and 2) introduction to the animal husbandry-based microfinance program, Pigs for Peace (PFP), under the direction and management of PAIDEK. The informational session will allow for village education, dialogue and questions related to microfinance and specifically, PFP. The outcome of the informational session will be the establishment of the PFP Village Association and leadership. Village Association membership is opened to villagers 25 years of age and older, regardless of gender. Membership in the Pigs for Peace Association is not limited to individuals who have reported or been identified as survivors trauma and violence for two critical reasons: 1) since the stigma and fear of rejection prevent many survivors of trauma and violence from reporting their cases or self-identifying as survivors, we do not want to limit access to services to women/men who have disclosed the violence; 2) rural women and men who are willing to invest time and resources in Pigs for Peace will be placed on the participation list for the loan of a pig. Further, engagement of village men in the associations will encourage their participation in the success of the project and their input on strategies to reduce stigma and isolation. Pigs for Peace village associations leadership committees, made up of men and women, will decide on the loan recipients. PAIDEK will work closely with the village associations to set up bylaws, membership rules, monthly meetings, loans and repayment, and monitor and collect data on outcomes.

*Village Household Recruitment:* In participating villages, the village leaders will work closely with the village based RAs, study coordinator and research team members to select household that meet the eligibility criteria: household with at least one member 16 years or older who is head of household, interest in animal husbandry microfinance, low-income, widow, survivor of sexual violence, children in the home. We estimate 66 households in each participating village will be eligible and consent to participate, for a total of 330 intervention households and 330 control households.

*Baseline Data Collection:* The study coordinator and village based RAs will visit the eligible households, invite them to participate and inform them of all study related activities. Participants in PFP are not required to participate in the study. If a PFP participant decides to not participate in the study, they will not be removed from the PFP project. Verbal informed consent will be obtained from each household participant prior to collecting baseline interviews. The interviews will be completed in a private setting, perhaps outside under a tree or in a quiet village location. The village based RA will complete the interview using a tablet computer that is pre-programmed with the questionnaire.. We estimate that each study interview will take one hour each. In our previous interviews, one hour was considered reasonable by the participants and did not cause undue burden.

*Study Interview Instrument:* The majority of the instruments have been used in prior work with internally displaced persons (IDPs) and refugees impacted by conflict. The measures have been used with diverse cultural groups in clinical and community settings. The measure includes demographics, quality of life, mental health, functioning, exposure to trauma and violence, family and community relationships, stigma and community support/reintegration.

Intervention Households

Within two weeks of the baseline individual and household member interviews being completed, the intervention household will be provided with the loan, one female pig. The loan recipient will be supported by the PFP village association to breed the pig, manage the health and care of the pig. The support comes through monthly association meetings as well as the study project coordinator providing regular home visits to help problem solve and assess success and challenges. The study village based RAs will complete follow-up face-to-face interviews that consist of the same measures as at baseline at 6,12 and 18-month post-baseline interviews. Eighteen months of follow-up was deemed necessary to provide time for the loan “pig” to successfully breed and have at least one litter. All village based data collection will be monitored and supervised monthly by the PI, co-investigators and coordinator. The team will meet monthly via Skype or, teleconference to discuss on going activities, challenges and successes. This communication strategy has been successfully implemented in previous studies.

*Intervention Monthly Association Meetings*: As part of PFP, each association member is expected to participate in monthly village based association meetings. Building on the success of the IMAGE study in South Africa,^14^ the purpose of the monthly meeting is two-fold: 1) In the first hour, the association members: a) discuss progress and challenges in PFP; and b) problem-solve challenges, like breeding needs, care for a sick pig, nutrition of pigs during pregnancy, health of piglets. The informative topics will be presented and led by village based research assistants who have received training in Year 1 as both research assistants and advocates for GBV victims, leadership and human rights.

*Intervention Fidelity*: Several techniques will be used to monitor intervention fidelity at each village location. The study partners will collaborate on the development of a training manual targeting the specific activities of the study. The training manual will describe the key elements of the intervention, delivery techniques, safety protocols and individual and household recruitment and retention protocols. The training will be conducted with investigators and RAs in Year 1. Ongoing supervision of RAs will be established. Supervision and monitoring will include monthly RA team meetings, monitoring/observation of randomly select RA contact with participants – including RA’s assessment of eligibility, consent procedures, and implementation of intervention protocols. Intervention fidelity will be included in the analysis plan by examining the intervention and monthly association sessions delivered and the protocol content delivered, compared to anticipated outcomes. Intention-to-treat analysis will be used to determine the extent to which subject loss or missing data may have impacted the study results. Intervention fidelity monitors will also document any changes in the study locations that may have influence the intervention, subtle changes to the delivery of the intervention over time, and if the subtle changes were associated with more or less positive outcomes for study participants. This monitoring plan provides the team with training by using a formal training program that includes experiential processes and includes quantitative evaluation of the fidelity of the training and delivery of the structured intervention.

*Process Evaluation*: Along with the individual and household measures collected at baseline and follow-up interview, a process evaluation of the PFP project performance in each intervention village will be completed at the end of 18 months with a sub-sample of association leaders and participants and include: 1) number of participants in village association; 2) number of pigs “loaned”; 3) health of pig and piglets; 4) repayment of loan piglets to association; 5) distribution of loan piglets to other members of association; 6) satisfaction with Pigs for Peace by association members; 7) opportunities to diversify loans, include rabbits and guinea pigs in the project. The findings from the process evaluation will be used to inform future large-scale implementation of the PFP program.

Delayed Control Households

Delayed control household procedures and data collection is consistent with that presented above in the intervention households. Control households will agree to participate in a monthly village association meeting. The control households will be eligible to receive the repayment of the loans from the intervention groups as consistent with the PFP model. The control households will receive their loan pig after the first year of the project.

Data Management and Analysis for Specific Aims 1 & 2

All research team members will successfully complete research training on responsible conduct of research (RCR) using the on-line Collaborative Institutional Training Initiative (CITI) prior to their involvement in the study. All research team members will receive and review documentation on policies and procedures for managing and reporting any emergency situations or adverse events that might arise during the study. An on-line training program for RAs is prohibitive, therefore the PI (Dr. Glass is able to speak French) will complete face-to-face training on RCR. The study investigators, who have many years of experience in the field of SGBV and intervention research, will train all village based research staff prior to their involvement in the study. The training will include sensitization to experiences of SGBV, maintaining the confidentiality of all research participants, intervention protocols and fidelity to protocols as well as issues related to informed consent and adverse events. All research staff members will receive a training manual (French and English) as a reference and resource.

*Data Management:* SPSS-PC software (version 18) will be used for data entry and analysis. The

Information will be exported from Microsoft Access to SPSS. Back-up data will be maintained on password protected USB drives. Each participant will be given a unique numeric code for all computer data records to maintain confidentiality. All data will be kept in locked offices and file cabinets and files will be protected with passwords.

*Pre-testing:* Prior to official recruitment, we will recruit a total of 15 households as pre-testers of the research process. The pre-testers will be asked to evaluate the interview questions, translation and understandability and content. The research protocol will be followed by the RAs and evaluated by them for use. The data collected will be evaluated for completeness and consistency. RAs will be evaluated for protocol accuracy. In the event that questions prove unclear or difficult to answer, the interview will be modified and retested.

*Missing Data:* Missing data are expected to be minimal based on our extensive pilot work. In the event

of household loss those households will be included in the analysis. Additionally, those households that drop out of the study will be compared to those who remained to determine any differences between the groups at baseline and at subsequent times for descriptive purposes and to determine if data is missing at random. Any variables that are related to the missingness will be included as covariates in the model and multiple imputations with chained estimation will be used to estimate missing data. We will conduct a residual analysis following the multilevel models to compare the residuals for models using completers only, all subjects without multiple imputation, and all subjects with multiple imputation to understand any biases introduced by missing not at random.

*Sample size calculation:*  We used data from Roberts and colleagues^56^ study of the reliability and validity of the SF-8 with a conflict-affected population in northern Uganda as the basis for our power analyses. We based the power analysis on repeated measures ANOVA, but propose to use multilevel modeling for the analyses that allows all cases with missing data to be included in the analyses. We do not have adequate estimates of the within and between variance to estimate power for the multilevel model directly. However, in general multilevel model approaches are more powerful than repeated measures ANOVA^62^. Since we do not have an estimate of the intraclass correlation (ICC) (individuals nested within villages) we varied the ICC from .001 to .010. For a sample size of 300 per group and assuming no change from baseline to 18 months in the control group and a 10% improvement in the intervention group, an alpha level of .05, the power to detect a significant group by time interaction for the physical health component score of the SF-8 Is .94, .89, and .83 for ICCs of .001, .005, and .010, respectively. Similarly power to detect a significant group by time interaction for the mental health component score of the SF-8 is .91, .84, and .78 for ICCs of .001, .005, and .010, respectively.

*Data Analyses:* All analyses will be based on intent-to-treat principles. Multilevel modeling will be used to test Specific Aim 1 to determine the effectiveness of a village-led microfinance program on general health, psychological health (depression, PTSD, suicidality) and reintegration (stigma, reintegration/acceptance by family) in intervention villages compared women in control villages. Time (baseline, 6, 12, and 18 months) will form the first level of the model and women the second level of the model. Logistic or normal distribution models will be used depending on the scale of measurement for the outcome variable. Variables related to missingness will be included in the second level of the model along with group (intervention vs. control) as an independent variable predicting change over time. A significant coefficient for group would imply that the pattern of change in the outcome variable differed for the intervention and control households. Separate analyses will be conducted for the target woman and second member of each household. Similarly, multilevel modeling will also be used to test Specific Aim 2 to determine the effectiveness of a village-led microfinance program on household (food security, children in school, utilization of health care services) economic stability in intervention households compared to control households. In these analyses, household will be the second level of the model.

Overview of Specific Aim #3.

To achieve specific aim 3, village residents (n=5 in each intervention/control village, total n=50) will complete a baseline and 18-month post-baseline in-depth qualitative interview with the village-based RA and/or study coordinator. The interview will provide an in-depth examination of the role of economic stability on village-level health, stigma and reintegration in both intervention and control villages.

*Recruitment and Data Collection;* The research team will work closely with village based RA to identify 5 village female and male residents in each participating village who are eligible (16 years or older) and willing to participate in two 90 minute in-depth interviews. The interviews will be completed at baseline and 18-months post-baseline and will examine their perspective on the impact of household economic security on health, stigma, and reintegration of survivors of trauma and violence into their village. The participants will not have been previously interviewed.

The purpose of interviews is to include a valid exploratory research technique to enrich the study activities and facilitate dialogue on health/stigma consequences of trauma and violence,, economic empowerment, reintegration of survivors. The findings will be used to further inform the intervention. Interviews are an excellent method for generating domain-specific content for the development of contextually-valid information that can inform existing and future interventions.^63^ Specifically, interviews discussions can indicate important content domains that need to be addressed in the culturally specific language of the participants. The interview also provides a safe, informal environment to learn from others’ experiences and insights. Trained research team members will conduct the interviews in a private village location. Prior to starting the interview, the team will administer an anonymous demographic questionnaire (e.g. age, gender, number of children and household members, household economic security, relationship status) to each participant. Participants will also be asked their permission to audiotape the individual interview. The interview discussions will focus on three key areas at the level of the village: 1) experience of trauma and violence; 2) stigma and health consequences of trauma and violence; 3) role of increased household economic stability in building social capital and social networks to address stigma, health and reintegration of survivors to the village. Participants will receive household goods (1kg rice, salt, soap) or small amount of money for time and expertise.

*Transcription and Translation:* The transcripts from the interviews will be prepared in the local language and then translated to French and English for use by all the research team members. Conducting the transcription in both the local language, French and English will allow for all members of the research team (local experts and academic partners) to participate in the analysis and compare transcripts during the analysis phase to enhance understanding or resolve questions in meaning and analysis.^8^ Additionally, the demographic questionnaire will be translated and back-translated in partnership with local experts.

*Qualitative Analysis:* The recordings from the interviews will be transcribed verbatim and saved electronically. Transcripts will be reviewed for accuracy by the team members who conducted the interviews prior to proceeding with the analysis. The analysis will follow a procedure to delineate common themes or generalizations about trauma and violence, stigma/health and role of economic stability on village health and well-being. The transcribed text of interview will be analyzed as follows: 1) the transcripts from interviews will be read in their entirety by two research team members to identify thematic codes relevant to key areas; 2) text segments will be coded according to the developed index codes. Examples of these index codes might follow along the lines of the interviews participants (gender, age, role in community, reintegration/exile from family and/or village), including types of stigma experienced by trauma and violence survivors as well as clarity and strength of responses to questions; 3) two research team members will collaboratively assign codes to corresponding sections of the text and discrepancies in coding will be discussed with the entire team. Such double coding of transcripts allows systematic monitoring of inter-coder reliability and maintenance of quality control. In addition to the thematic analysis, key domains of the interview findings and additional domains suggested by experts will be examined to strengthen the intervention.

*Authenticity and Trustworthiness of Qualitative Data and Analysis*: Qualitative interpretation requires implementation of safeguards to assure credibility, confirmability, and authenticity.^64, 65^ Credible interpretation must be a good ‘fit’ between the respondents’ views and experiences and the researcher’s interpretation of The interpretation process must be sound and relatively free of biases during the research process. Member checking is an available tool in this analysis, a sub-sample of interview participants will be selected to review the findings and discuss needed revisions.^64, 65^ Regarding confirmability, the research team will engage in peer review and debriefing during the analysis process as a qualitative mechanism that serves a similar function to inter-rater reliability in quantitative research.^63^ Specifically, during the iterative coding process, each of the research team members will read the transcripts first and draw individual conclusions. The team will come together and discuss findings and interpretations as necessary during the analysis process to assure consistency in interpretation.

## Importance of knowledge gained and potential benefits of the proposed research to the participants and others

Trauma and violence is a global health issue and is a violation of human rights and has been used as a weapon of war in the DRC. The multiple interrelated and complex social determinants of health, such as poverty, stigma, chronic stress and trauma, and limited access to health care services impacts the health of individuals, their household and village. As a major donor to global health and humanitarian crisis, the US needs improved evidence of sustainable community-led intervention models for improved health outcomes to reduce health disparities in low-income countries. Research findings from the three study aims will inform the science base for large-scale implementation of structural interventions, with the aim to establish a causal relationship between a village-led microfinance intervention and increased health, household economic stability, and reintegration for survivors of trauma and violence. Further, the study will provide the basis for the transferability of the intervention to other settings, including man-made and natural disaster settings.

Table 2: Study Timeline

| **Activity** | **Year 1** | | | | **Year 2** | | | | **Year 3** | | | | **Year 4** | | | | **Year 5** | | | |
| --- | --- | --- | --- | --- | --- | --- | --- | --- | --- | --- | --- | --- | --- | --- | --- | --- | --- | --- | --- | --- |
|  | Q1 | Q2 | Q3 | Q4 | Q1 | Q2 | Q3 | Q4 | Q1 | Q2 | Q3 | Q4 | Q1 | Q2 | Q3 | Q4 | Q1 | Q2 | Q3 | Q4 |
| IRB approval/study set-up/training of RAs/village consent | X | X | X | X |  |  |  |  |  |  |  |  |  |  |  |  |  |  |  |  |
| Recruitment/retention (Household) |  |  |  | X | X |  |  |  |  |  |  |  |  |  |  |  |  |  |  |  |
| PFP Intervention households |  |  |  |  | X | X | X | X | X | X |  |  |  |  |  |  |  |  |  |  |
| Delayed control households (association meetings) |  |  |  |  | X | X | X | X | X | X |  |  |  |  |  |  |  |  |  |  |
| Delayed control households (intervention) |  |  |  |  |  |  |  |  |  |  | X |  |  |  |  |  |  |  |  |  |
| Follow-up contacts (intervention/control) |  |  |  |  |  | X | X | X | X | X | X | X | X | X | X | X |  |  |  |  |
| Retention follow-ups (intervention/control) |  |  |  |  |  | X | X | X | X | X | X | X | X | X | X | X |  |  |  |  |
| Fidelity Monitoring |  |  |  |  |  | X |  | X |  | X |  | X |  | X |  | X |  | X |  |  |
| Qualitative Interviews (intervention/control households) |  |  |  |  | X |  |  |  |  | X | X |  |  |  |  | X |  |  |  |  |
| DSMB meets |  | X |  | X |  | X |  | X |  | X |  | X |  | X |  | X |  | X |  | X |
| Data entry |  |  |  | X | X | X | X | X | X | X | X | X | X | X | X | X | X | X |  |  |
| Data Cleaning |  |  |  |  |  |  |  | X |  |  |  | X |  |  |  | X |  | X |  |  |
| Data Analysis |  |  |  |  |  |  |  |  |  |  |  | X | X | X | X | X | X | X | X | X |
| Annual Reports |  |  |  | X |  |  |  | X |  |  |  | X |  |  |  | X |  |  |  | X |
| Manuscripts & Dissemination |  |  |  | X |  |  |  | X |  |  |  | X |  |  |  | X |  |  | X | X |
|  |  |  |  |  |  |  |  |  |  |  |  |  |  |  |  |  |  |  |  |  |

Q1=Sept-Nov; Q2=Dec-Feb; Q3=March-May; Q4=June-August

**Study Partners: GREAT LAKES RESTORATION**

Great Lakes Restoration (GLR) is a non-profit organization with the goal of “Building the Peace” by assisting the poor people in the Eastern African countries of the Great Lakes region, primarily the Democratic Republic of the Congo (DRC), Rwanda, Burundi, Kenya, Uganda, and Tanzania, in the post-conflict recovery from recent warfare, through the development of the human capital capacity. The projects sponsored by GLR are designed to stimulate creativity, work, ambition and hope. These are not “quick-fix” projects but are designed to establish the long term future growth, peace, and stability in the Great Lakes region.

Our projects, our “buildings”, will address the following components inherent in
“Building the Peace”:

- INFRASTUCTURES to improve the living conditions of the population and
  effectively fight poverty.
- EDUCATION to promote the basic tools necessary for growth.
- HEALTH to provide the population with the services and medicine needed to combat disease, poor nutrition, HIV/AID, and premature death.
- WATER and ELECTRICITY which are essential to sustain human life in the modern world.
- HOUSING to promote human dignity through improved living conditions.
- EMPLOYMENT to provide hope and an alternative future for people to live and prosper in peace.

Dr. Cinyabuguma, Study Consultant, is the founder and genesis of the Great Lakes Restoration (GLR). He was born in the Democratic Republic of the Congo (DRC) and grew up in the Bukavu area in South-Kivu Province. He is currently an Assistant Professor of Economics at University of Maryland-Baltimore County (UMBC) where he teaches Macroeconomics and Mathematical Economics. Since 2007 Dr. Cinyabuguma has been the designated representative of the Catholic University of Bukavu (UCB) in America and in 2008 he was elected to the Board of Trustees. Before coming to UMBC, he was a Lecturer in Economics and Statistics at UCB for three years. Dr. Cinyabuguma has consulted with the IMF; the World Bank; the Center for Experimental Economics at Brown University; the Central Bank of the DRC; Gecamines Commerciale (DRC); and, various NGOs in the DRC and others international in scope. He chairs the board of directors of an NGO, International Agency for Refugee Movement (IRAM) that is working to improve the lives of refugees around the world. Among his many publications is “Sources of Growth in the Democratic Republic of the Congo: A Co-integration Approach”, jointly with Akitoby Bernardin, in Post Conflict Economics in Sub-Saharan Africa: Lessons of the Democratic Republic of the Congo, 2004, ed. Jean A.P. Clement, International Monetary Fund. His current research is concerned with analyzing the impact of institutions and corruption on economic development using theoretical and empirical models; studying fertility and economic growth; and understanding behavioral economics using lab experiments. Given his current research in international development and his social background, Dr. Cinyabuguma believes that education -- taken in the very broad sense of teaching people, especially the young, how to live in peace and prosperity -- is the best path towards human development and poverty reduction in poor countries around the world. Because of this fundamental belief of Dr. Cinyabuguma, he has formed a team at GLR whose focus is to further the cause of education for all and to empower the young especially (The median age of the population of the DRC is sixteen (16).) in the fight against some of the threats, such as warfare, disease and poverty, to the lives of the people of the Great Lakes region.  This idea, then, of using education to drive development is the essence of human capacity development and of GLR programs. Unlike other organizations, GLR will focus on endogenous development, utilizing internal resources, human and otherwise, in so far as possible, with projects that advance human capacity building with direct bearing on the lives of the people of the region.  Given this orientation, GLR partners with the University Catholic of Bukavu (UCB), and its Chancellor, Monsignor Xavier Maroy, who is also the Archbishop of Bukavu and with the Conseil Regional des Organisations Non Gouvernementales de Developpement du Sud-Kivu (Regional Council of Non-Governmental Development Organizations of South Kivu) (CRONGDSK) and Johns Hopkins University School of Nursing and Medicine in implementing its programs. Dr. Robert Bollinger, Professor of Medicine at the Johns Hopkins University School of Medicine and Associate Director, Hopkins Center for Global Health serves as the Chairman of the GLR Advisory Board. GLR maintains an office in Warrenton, Virginia. The office provides coordination for ongoing collaboration between US (Hopkins among others) and Congolese partners, such as PAIDEK that are working towards peace and development in the Great Lakes region of Africa. Dr. Cinyabuguma will not have access to identifiable data during the study.

**Study Partners: PAIDEK MICROFINANCE**

PAIDEK will implement the microfinance intervention, Pigs for Peace, but will not be involved in collecting evaluation data for the study. PAIDEK is a non-profit, non-governmental organization founded in 1995 in the Democratic Republic of Congo (DRC). It was organized pursuant to the laws of the DRC and was legally recognized on May 3, 2005 by the Provincial Division of the Ministry of Justice as a legal entity by order No. JUST.G.S. 112/S-KV/1980/2005. PAIDEK has maintained operations and programs throughout the 15 years of war in Eastern DRC. PAIDEK’s purpose is to create conditions supportive to development and reviving the economy of the South and North Kivu Regions of Eastern DRC. PAIDEK’s focus is on financial support for microeconomic development initiatives in the region, targeting individuals and villages that do not have access to traditional banking or credit institutions. In pursuit of this goal PAIDEK has expanded to eight (9) offices located throughout North (Goma, Butembo and Beni) and South (Bukavu, Idjwi, Kadutu, Katana and Uvira) Kivu provinces in the eastern DRC, with the headquarters located in Bukavu, South-Kivu. There are 36 employees who manage PAIDEK’s activities at the various locations. Each office is equipped with 2 desktop computers and a printer. All 9 offices have internet access. PAIDEK also maintains 5 laptop computers and 3 printers for travel of staff. Each employee has a mobile phone for work-related activities.

Projects and activities for PAIDEK are overseen by Mitima Remy as Director of PAIDEK (key contributor to the proposed study). If funded, Mitima Remy will be responsible for hiring the coordinator and field RAs and provide ongoing supervision to the PAIDEK staff in their work and study related activities with of PFP village associations and participating families.

Currently PAIDEK has over $2,000,000 in loans placed supporting a wide variety of enterprises. These activities range from purely microfinance funding for individuals and other entities to providing a wider spectrum of support, including management and marketing for initiatives in areas such as crafts manufactures and agriculture, such as Pigs for Peace (PFP), the animal husbandry microfinance program serving rural families. As with PFP, many of the other microenterprises PAIDEK supports have cross-cutting goals promoting social and development progress beyond their economic benefits.

PAIDEK’s initiatives are conducted through customer or client entities located throughout South and North Kivu Provinces in Democratic Republic of Congo. These entities can be as small as an individual or a family. As of the present time over 13,000 such customer entities have been or are clients of PAIDEK. On average each entity is composed of or benefits seven (7) family members. The average family size is seven (7) in this region of DRC. This means that 91,000 people have and are benefiting from capital in excess of $2,000,000 that PAIDEK has invested in its clients varied enterprises. The average loan is $350 with a 1-3% interest rate. Repayment of loans is extremely high, over 90% of loans are paid in full within the loan period (average 3 months). PAIDEK’s web presence may be found at: <http://www.sosfaim.be/pages_be/en/partenairesSud/be_en_partenaires_congokin_paidek.html> and the email address is [paidek@hotmail.com](mailto:paidek@hotmail.com).
